# Prevalence of post-partum anemia and associated factors among women attending public primary health care facilities: An institutional based cross-sectional study

**DOI:** 10.1371/journal.pone.0263501

**Published:** 2022-02-03

**Authors:** Alex Mremi, Doris Rwenyagila, Joseph Mlay

**Affiliations:** 1 Department of Pathology, Kilimanjaro Christian Medical Center, Moshi, Tanzania; 2 Facutly of Medicine, Kilimanjaro Christian Medical University College, Moshi, Tanzania; 3 Department of Obstetrics and Gynecology, Kilimanjaro Christian Medical Centre, Moshi, Tanzania; 4 Department of Obstetrics and Gynecology, Muhimbili University of Health and Allied Sciencies, Dar es Salaam, Tanzania; Waikato Institute of Technology, NEW ZEALAND

## Abstract

**Background:**

Severe post-partum anemia is an important cause of maternal deaths and severe morbidity in sub-Saharan Africa. In Tanzania, little information is available to guide health care professionals in ensuring good health of women after delivery. The objective of our study was to determine the prevalence of post-partum anemia and associated factors among women attending public primary health care facilities.

**Materials and methods:**

An institutional based cross sectional study was carried out. Women in post-partum period (the period from child birth to six weeks after delivery) attending the public primary health care facilities from October to December 2019 for children vaccination were recruited. The prick method was used to obtain blood for haemoglobin estimation. Post-partum anemia was defined as a haemoglobin level of less than 11g/dl. Participants found anaemic were asked to undertake malaria and helminths parasites tests from blood and stool samples respectively. The samples were examined by an experienced laboratory scientist on study sites according to the Tanzania national standard for medical laboratories protocols.

**Results:**

A total of 424 women were enrolled with mean age of 27.8 years (SD 5.93). Most of the participants 234(55.2%) had primary education and nearly half 198(46.7%) of them were house wives. The overall prevalence of post-partum anemia was 145(34.2%). Among the anaemic participants, 34(23.5%) had positive blood slide for malaria parasite while 15(10.3%) had positive test for stool helminths infection. Delivery by vaginal route and low parity were protective against post-partum anemia (p<0.001).Other factors that were associated with post-partum anemia included absence of a marital partner (p<0.001) and inter pregnancy interval of less than two years (p<0.001). The risk of post-partum anemia in women with less than two years interval between their last two pregnancies was about 18 times more as compared to women with more than two years interval between their last two pregnancies, (COR = 18; 95% CI 8.617–38.617).Women without marital partners were 10 times more likely to get anemia as compared to married women, (COR = 10; 01.910–54.935).

**Conclusions:**

The prevalence of anaemia among post-partum women found in this study points to a situation of public health problem according to WHO cut-off values for the public health significance of anaemia. Inter pregnancy interval of less than two years and absence of a marital partner were associated with post-partum anemia while delivery by vaginal route and low parity were protective against post-partum anemia. Strategies should therefore be put in place to encourage thorough health education and promotion programs among both pregnant and post-partum women.

## Introduction

Post-partum anemia is a common problem throughout the world and the prevalence is high in developing countries where it ranges between 50% and 80% [1]. Post-partum period begins immediately after the delivery of the placenta and lasts up to 42 days. It is the most critical period for the health and survival of both the mother and her neonate [1]. Post-partum anemia (haemoglobin level of less than 11g/dl) is one of the commonest puerperal complications and major cause of maternal morbidity and mortality [2]. In developing countries, the most important causes of post-partum anemia are prepartum iron deficiency and/or iron deficiency anemia combined with acute bleeding anemia at delivery, infections such as malaria, intestinal worms; disease conditions such hemoglobinopathies, poor socio-economical conditions and nutritional deficiencies [1–4]. Other related factors include education level, income, age, parity, birth spacing, antenatal care, blood loss and delivery complications [5]. Post-partum haemorrhage and pre-partum anemia may also cause postpartum anemia in 5% to 25% [5]. While anemia has been studied extensively in pregnant women, the extent and impact of anemia during the postpartum period has more recently become an area of interest [6].

The prevalence of anemia in Tanzanian women during pregnancy and after delivery is unacceptably high. A cross-sectional study conducted in a capital city, Dodoma in 2020 found the prevalence of postpartum anemia to be 21.6% [7]. A study on determinants of anemia in postpartum HIV-negative women in Dar es Salaam found that at baseline, 67.4% of the population was anemic [4]. Maternal nutritional status during pregnancy, prenatal iron/folate supplementation, perinatal care, and prevention and management of infections, such as malaria, were modifiable risk factors for the occurrence of, and recovery from, anemia [4, 7, 8].

Anemia during pregnancy is associated with adverse maternal health, possibly contributing to maternal mortality, and with poor pregnancy outcomes, including low birth weight, premature birth, neonatal and infant morbidity, and mortality, as well as reduced newborn iron stores and subsequent cognitive impairments [7]. The mother’s health status during pregnancy and postpartum period affects the potential for breastfeeding, and consequently the risks of anemia in infancy [1]. This is more apparent in mothers below the poverty level, including those from developing countries.

Severe pre-partum anemia has a negative impact to both maternal and child health, as it is associated with increased risk post-partum haemorrhage, depression, reduced cognitive functions and urinary tract infection, fatigue and exhaustion, insufficient milk syndrome and reduced breast milk quality [7–10]. Severe anemia may also leads to delayed wound healing, reduced immunity, increased susceptibility to breast infection like mastitis, ductitis, and hence has major impact on maternal and child’s health [11]. Previous studies have also shown that recovery from anemia after delivery was slower for low-income women relative to those with family income above the poverty line [6, 12].

Studies on the burden of post-partum anemia and associated risk factors among women in Tanzania are limited. Understanding the risk factors associated with post-partum anemia may help provide valuable information that can be useful in the intervention strategies to reduce anemia and its complications. This study aimed at determining prevalence and factors associated with post-partum anemia among women attending public primary health care facilities in Dar es Salaam, Tanzania.

## Materials and methods

### Study design

This was an institutional-based cross-sectional study conducted over a period of three months prospectively from 1^st^ of October, 2019 to 31^st^ of December 2019 among post-partum women attending Maternal and Child Health clinic in primary public health care facilities.

### Study setting

The study was conducted in three public health centres namely Mnazi Mmoja, Kigamboni and Magomeni in Dar es Salaam; the largest city in Tanzania with a population of about seven million [13]. Dar es Salaam city was selected because it has the highest number of health care facilities compared to other regions of Tanzania. The city has other hospitals including the National hospital (Muhimbili National Hospital). The three health facilities were purposely and conveniently selected to represent other public healthcare facilities in the city. Primary health care facility is the first place of contact regarding the issue of prevention and treatment in the community. Primary health care is available for large part of the citizens and is the main link to other advanced health care services in the country. The study was conducted in reproductive and child health (RCH) clinics. RCH clinics provide maternal health services, neonatal and child health services, family planning services, adolescent health and sexual transmitted diseases. Maternal health services provided include essential obstetric care, emergency obstetric care, and safe abortion services. Neonatal and child health services which are provided include vaccinations, micronutrient supply, etc. Family planning services include education, counselling and HIV testing and provision of a family planning methods. Postnatal care includes general and physical examination of the mother and her infant, education regarding danger signs, hygiene, importance of breastfeeding, etc. Within these health facilities mother and child clinics serve for baby vaccination and review of the mothers.

### Study population

Study population included women who delivered within six weeks during the study period. The entry point was the under-fives vaccination clinics of the identified health centres from 1^st^ of October 2019 to 31^st^ of December 2019.The measurable parameters together with data collection were done at that point in time.

### Eligibility criteria

#### Inclusion criteria

Included into the presented study were biological mothers who delivered within six weeks time during the study period attending vaccination program for their infants; and were willing to participate in to the study.

#### Exclusion criteria

Women who delivered more than six weeks time, those with post-partum depression or physical conditions that prevented free participation into the study and those unwilling to participate in the study were excluded from the study. Further, we excluded women with chronic illness or conditions that could lead to anemia such malaria, active helminthes or other parasitic infections, hemoglobinopathies, malignancies, tuberculosis etc; using the checklist as well as the self-reported awareness of these the conditions.

#### Research variables

Dependent variable was post-partum anemia while the independent variables were age, parity, education, marital status, occupation, inter-pregnancy interval, HIV status, mode of delivery, pre-existing anemia, history of post-partum hemorrhage (PPH) and multiple gestations.

#### Sample size estimation

Considering the prevalence of post-partum anemia of 42% [4] with a 95% confidence interval, absolute precision of 5% and assuming a non-response rate of 10%. The total adjusted sample size estimation using the Leslie formula for sample size of proportion in a large population was 404 women.

#### Sampling procedure

Convenient sampling was done for all women attending vaccination programs for their infants, until the sample size was obtained. Three research assistants (two experienced nurses and one laboratory scientist) at each of the three health centres were identified and trained by the principal investigators (PIs) on the study objectives and protocol. After qualifying, the research assistants assisted the PIs in data collection. The recruitment of study participants was conducted mainly in the vaccination rooms and few from postnatal clinics.

#### Data collection and ennoblement procedures

Data was collected using a proposed checklist. Collected data included the name of the health facility, patients’ registration number, social demographic characteristics such as age of the participants, marital status, maternal education, spouse education and occupation, child spacing and parity these were obtained from the participants. Obstetric characteristics such as mode of delivery, history of multiple gestations, estimated blood loss, prenatal haemoglobin, iron and folate supplementations (participants were only asked if they received the supplements or not regardless of duration of utilization), malaria prophylaxis (SP), deworming with mebendazole, booking time for antenatal clinics and number of antenatal care (ANC) visits were obtained from antenatal cards of the participants. History of medical conditions such as hypertensive disorders, hemoglobinopathies, malignancies, tuberculosis and other conditions that may lead to anemia were also recorded.

#### Haemoglobin measurements and other laboratory tests

Haemoglobin measurements of blood samples from post-partum women were performed by using HamoCue hematologic analyzer by an experience laboratory scientist at the study sites. The analysis was performed immediately after the samples were collected. Briefly the procedure involved the disinfection of the middle finger of the participant and allowed it to dry and then pricked at the side of finger tip. The drop was drawn and placed onto a micro cuvate which was then inserted into the haemo-analyzer and a reading was obtained within 45 seconds. The value was recorded and disclosed to each participant. Participants who had haemoglobin levels of less than 11g/dl were considered anemic thus, were asked to undergo further investigation for the presence of malaria and helminths parasites in the blood and stool respectively. Once the blood samples for malaria parasite and stool samples for helminths were collected; the samples were immediately examined by an experienced laboratory scientist at respective study sites according to the Tanzania national standard for medical laboratories [14].

### Data management

Data collected in the study were entered in duplicate in IBM SPSS version 20 statistical program. Data was checked for missing data and consistency. Data analysis was done to determine the strength of association between dependent and independent variables using Chi-square for categorical variables. Categorical variables were summarized using proportions. All statistical tests were performed at 5% significance level (95% confidence level) and summarized data were presented in tables. A logistic regression model was fitted to the anemia data to explain the predicted odds of post-partum anemia. Inclusion of predictors in the model was based on statistically significant association (p value <0.001) in the bivariate analysis.

### Ethical consideration

Ethical clearance was sought from the Research and Publications Committee of the Muhimbili University of Health and Allied Sciences (Clearance # 80). Permission was also obtained from the management the District’s Medical Officers of the respective municipals. The purpose and the importance of the study were explained to each study participant prior to interview and data collection. Written informed consent to participate into the study was obtained from each woman. Privacy and confidentiality were assured by removal of identifiers and each participant was interviewed individually. Women who were found to be anemic and those who had positive for malaria and helminths infections were treated according to facility protocols.

## Results

A total of 447 post-partum women were assessed for eligibility into this study and 23 did not meet the criteria thus, were excluded. The reasons for exclusion were; currently taking iron supplements and or anti-parasitic medication for malaria and or helminthiasis (n = 9), history of malignancy/pre-malignancy (n = 7), hemoglobinopathies (n = 4), and not willing to participate into the study (n = 3). Thus, a total of 424 postpartum women were recruited in this study. Each facility contributed about a third of the participants. Their mean age was 27 years (SD 5.93). Majority 338 (79.7%) of the participants aged between 20 and 34 years; 234(55.2%) had primary education and nearly half 198(46.7%) of them were house wives. Of 424 enrolled women, 145 (34.2%) had anemia (HB level of less than 11g/dl). The parity and marital status showed statistical significant association with post-partum anemia (p<0.001), Table 1. Majority 179(42.2%) of the study participants were having three or more parities. Most women 340(80.2%) booked antenatal care at the second trimester and 244(57.6%) had three to four visits, Table 2. Among obstetric factors evaluated, pregnancy interval between the last two pregnancies and mode of delivery in the last pregnancy were significantly associated with post-partum anemia (p<0.05), Table 2.

**Table 1:** Relationship between post-partum anemia and socio-demographic characteristics of women attending Reproductive and Child Health clinics at Mnazi Mmoja, Kigamboni and Magomeni Health Centers (N = 424).

Social demographics	N (%)	Anemia n = 145(%)	Non-anaemia n = 279(%)	*P*- value
Age Groups				
<19	20(4.7)	10 (6.9)	10 (3.6)	0.298
20–34	338(79.7)	114 (78.6)	224 (80.3)	
34+	66(15.6	21 (14.5)	45 (16.1)	
Education level				
Primary	234(55.2)	80 (55.2)	154 (55.2)	0.596
Secondary	166(39.1)	59 (40.7)	107 (38.4)	
College	24(5.7)	6 (4.1)	18 (6.4)	
Marital Status				
Single	53(12.5)	40 (27.6)	13 (4.7)	0.001
Married	371(87.5)	105 (72.4)	266 (95.3)	
Occupation				
House wives	198(46.7)	65 (44.8)	133 (47.7)	0.230
Employed	67(15.8)	29 (20.0)	38 (13.6)	
Business	159(37.5)	51 (35.2)	108 (38.7)	
Parity				
1	130(30.7)	26 (17.9)	104 (37.3)	0.001
2	115(27.1)	19 (13.1)	96 (34.4)	
3+	179 (42.2)	100 (69.0)	79 (28.3)	
Health facility				
Kigamboni	141(33.2)	42 (29.0)	99 (35.5)	0.367
Mnazi Mmoja	142(33.5)	50 (34.5)	92 (33.0)	
Magomeni	141(33.2)	53 (36.5)	88 (31.5)	
HIV Sero Status				
Positive	37(8.7)	14 (9.7)	23 (8.2)	0.374
Negative	387(91.3)	131 (90.3)	256 (91.8)	
Medical Conditions				
Yes	6(1.4)	2 (1.4)	4 (1.4)	0.664
No	418(98.6)	143 (98.6)	275 (98.6)	

Medical conditions: Hypertension/Diabetes mellitus/Obesity.

Anemia: HB<11 g/dl; Non-anemia: HB≥11g/dl.

**Table 2:** Relationship between anemia and obstetric characteristics of women who attended Reproductive and Child Health clinics at Mnazi Mmoja, Kigamboni and Magomeni Health Centers (N = 424).

Obstetric history	Anemia n = 145 (%)	Non -anemia n = 279(%)	N = 424(%)	P value
Interval between the last pregnancies				
2 years or less	105 (72.4)	26 (9.3)	131(30.9)	0.001
>2 years	40 (27.6)	253 (90.7)	293(69.1)	
Mode of delivery				
SVD	97 (66.9)	243 (87.1)	340(80.1)	0.001
CS	48 (33.1)	36 (12.9)	84(19.9)	
Complication during/ after delivery				
Perineal tear	26 (17.9)	50 (17.9)	76(17.9)	0.689
Hemorrhage	9 (6.2)	12 (4.3)	21(5.0)	
No complication	110 (75.9)	217 (77.8)	327(77.1)	
Multiple births at last pregnancy				
Yes	3 (2.1)	8 (2.9)	11(2.6)	0.447
No	142 (97.9)	271 (97.1)	413(97.4)	
Antenatal clinic booking time				
1^st^ trimester	18 (12.4)	52 (74.3)	70(16.5)	0.137
2^nd^ trimester	120 (82.8)	220 (64.7)	340(80.2)	
3^rd^ trimester	7 (4.8)	7(50.0)	14(3.3)	
Antenatal number of visits				
<2	26 (17.9)	34 (12.2)	60(14.1)	0.238
3–4	82 (56.6)	162 (58.1)	244(57.6)	
4+	37 (25.5)	83 (29.8)	120(28.3)	

CS: Cesarean Section delivery, SVD: Spontaneous Vaginal Delivery.

Anemia: HB<11 g/dl; Non-anemia: HB≥11g/dl.

Table 3 summarizes utilization of key interventions for preventing anemia during antenatal and postpartum period. Majority of the participants 406(95.8%) received iron and folic acid (IFA) tablets while 378(89.2%) were dewormed during pregnancy. Most of the participants received between two 113(26.7%) and three 131(30.9%) doses of malaria prophylaxis (SP); and majority of them 418(98.6%) did not use insecticide treated nets (ITNs). Among antenatal interventions provided for prevention of anemia, among participants who utilized ITNs, none of them had anemia. At the same time the association between other preventive measures and risk of post-partum anemia did not attain any significant association, Table 3.

**Table 3:** Relationship between utilization of Interventions for prevention of anemia during pregnancy and postpartum period at Mnazi Mmoja, Kigamboni and Magomeni Health care facilities (N = 424).

Intervention	Anemia n = 145(%)	Non Anaemic n = 279(%)	N = 424(%)	P value
Insecticide treated nets				
Yes	0 -	6 (2.1)	6(1.4)	0.081
No	145 (100)	273 (97.9)	418(98.6)	
Iron and folic acid supplementation				
Yes	138 (95.2)	268 (96.1)	406(95.8)	0.668
No	7 (4.8)	11 (3.9)	18(4.2)	
Mebendazole				
Yes	129 (89.0)	249 (89.2)	378(89.2)	0.525
No	16 (11.0)	30 (10.8)	46(10.8)	
Malaria chemoprophylaxis				
None	21 (14.5)	45 (16.1)	66(15.6)	0.393
1 dose	34 (23.5)	51 (18.3)	85(20.1)	
2 doses	37 (25.5)	76 (27.2)	113(26.7)	
3 doses	47 (32.4)	84 (30.1)	131(30.9)	
4 doses	6 (4.1)	23 (8.2)	29(6.8)	

Anemia: HB<11 g/dl; Non-anemia: HB≥11g/dl.

Nearly one quarter (23.5%) of participants with postpartum anemia had positive blood slide for Malaria falciparum parasites, while 15(10.3%) of them had helminthiasis, Table 4. Post-partum women with less than two years interval between the last two pregnancies were about 18 times likely to have anemia than women with more than two years interval, (COR = 18; 95% CI 8.617–38.617), Table 5. Single women were 10 times more likely to get anemia compared to married women. (COR = 10; 01.910–54.935). The risk of post-partum anemia was decreased (COR = 0.2; 95% CI 0.084–0.418) in women who delivered per vaginally as compared to those delivered by cesarean section. The risk of anemia was decreased in women with low parity compared to those with high parity (COR = 0.3; 95% CI 0.147–0.652), Table 5.

**Table 4:** Helminthiasis and Malaria falciparum parasitisation among anemic women attending primary public health centers in Dar es Salaam (n = 145).

Parasite	N = 145(%)
Helminths infestation	
Positive	15 (10.3%)
Negative	130 (89.7%)
Blood slide for Malaria falciparum parasite	
Positive	34 (23.5%)
Negative	111(76.5%)

**Table 5:** Multivariate regression analysis in relation to post-partum anemia among post-partum women.

Variable	N = 145(%)	COR	95% CI	P value
Interval between last two pregnancies				
Less than two years	107(73.8)	18		0.000
More than two years	38(26.2)	1	8.617–38.617	
Parity				
1	26(17.9)	0.3	0.147–0.652	0.002
2	19(13.1)	1		
3+	100(69.0)	1		
Mode of delivery				
Vaginal delivery	97(66.9)	0.2		
Cesarean section	48(33.1)	1	0.084–0.418	0.000
Marital status				
Single	40(27.6)	10	1.910–54.935	0.007
Married	105(72.4)	1		
Insecticide treated nets				
Yes	0(0)	1	0.001	0.9
No	145(100)	1		

COR: Crude Odds Ratio.

## Discussion

Prevalence of post-partum anemia in our study was 34.2%. This study highlighted that post-partum anemia is a significant public health problem in Tanzania. It has also recognized several factors associated with post-partum anemia such as delivery by cesarean section, child spacing, absence of a marital partner and increased parity of which have policy implications.

In the current study the prevalence of anemia during post-partum period was much lower compared to a study done in Uganda which reported the prevalence of anemia in nearly two thirds of the participants [15]. Such a big difference may be due to variation in hemoglobin cut- off points used to define post-partum anemia. However, the prevalence of anemia in this study is higher compared to the studies done from high income countries [16]. Advancements in health facilities and maternal living conditions in the western countries as compared to Tanzania could explain the observed differences. However, the high prevalence of post-partum anemia observed in our study highlights a need for further improvement in maternal nutrition and the general health of the women before and during pregnancy as well as post-partum period. Over the years, Tanzanian government through the Ministry of Health has been trying to strengthen antenatal care (ANC) services across the country. Currently, every pregnant woman has free access to iron supplementation to combat anaemia, deworming agents, malaria prophylaxis, and mosquito nets [17, 18].

In the current study, delivery by vaginal route was protective against post-partum anemia and the risk of post-partum anemia decreased in women who delivered per vaginally as compared to those delivered by cesarean section. The exact reason for this observation is obscure. Perhaps high risk of post-partum hemorrhage and hemorrhage related morbidity in cesarean delivery group may have resulted into higher prevalence of post-partum anemia compared to vaginal delivery, [19, 20]. This finding reminds the health care providers to take preventive measures against complications of post-partum anemia. Such prevention measures include screening, prophylaxis with iron tablets and treatment.

The inter pregnancy interval was significantly associated with post-partum anemia in the current study. The risk of post-partum anemia was found to be 10 times when the interval is less than two years between the last two pregnancies as compared with those with more intervals. This finding is consistent with previous reports [21, 22]. This is likely due to the fact that mothers may not yet replenished essential nutrients especially iron and folic acid which were depleted by the previous pregnancy. In this study, maternal age was not found to be significantly associated with post-partum anemia. Other investigators have also found no correlation between anemia in post-partum period and maternal age [19, 20]. However, younger maternal age has been implicated in the development of post-partum anemia because this age group tends to have incomplete growth, lesser utilization of prenatal and postnatal services and psychological factors [23].

Low parity was found to be protective against post-partum anemia in our study. Parity or number of previous pregnancies has been shown to impact the long term health status of women specifically anemia. The women with more than two pregnancies had significantly higher rate of anemia. Increasing parity has also been established to be associated with post-partum anemia by previous investigators [1–5]. This might be due to increase of women’s nutritional demand during pregnancy. Women with prior pregnancy sustain between 500mg and 600mg of iron loss per pregnancy which includes addition to daily iron loss in that part of gestation. Iron requirement directed towards the fetus and the placenta (300mg to 350mg) and puerperal blood loss (200mg to 250mg). Blood loss during delivery will also increase this loss. Anemia is therefore much more common as parity increase.

Our study has highlighted that women with no formal marital partners were more likely to get anemia as compared to married women. A study done in Ghana found no association between marital status and post-partum anemia [5]. The reason for the association found in the present study is not clear. Perhaps it was by chance. However, lack of consistent and reliable moral and socio-economical support from the male partner who is essential for health pregnancy may explain this observation. Women without permanent marital partners are likely to be financially unstable especially during pregnancy when their ability to engage in income generating activities is compromised and thus vulnerable to poor living conditions leading to poor nutrition in this group as compared to the married women [24, 25].

In the current study it was observed that 89.2% and 96.1% of the total study participants consumed hematinics and iron supplements respectively during pregnancy. There was a reason to believe that women’s lack of nutritional inputs predates post-partum anemia. It has been seen that despite of iron and folic acid and anti-hematinics consumption, post-partum anemia is still prevalent in this population. Perhaps women had high levels of nutritional deficiency prior pregnancy so that the supplemental iron was not enough to correct anemia. On the other hand women reporting consumption of hematinics might not be consuming them in reality and this needs further researching [26].

Although our study did not find any statistical significant association between booking time for ANC and post-partum anemia, other researchers have clearly established a positive correlation between late ANC booking and postpartum anemia. Early ANC plays a major role in preventing anemia [7, 23]. This is due to failure to benefit from chemoprophylaxis and late diagnosis and treatment of anemia among women who delay in utilizing ANC services. Therefore, in line with ANC guidelines by the World Health Organization as well as the Ministry of Health, Community Development, Gender, Elderly and Children; programs aiming at early ANC booking are highly emphasized. Women should attend ANC as soon as they find out that they are pregnant [17, 18].

The prevalence of malaria and helminthes parasite infections among participants in the current study is considerably high, despite the reported high utilization of preventive treatment during pregnancy. Our results agree well with previous study [27] in Tanzania which reported a significant correlation between anemia with malaria. A study from Kenya reported the prevalence of geohelminths, malaria parasites, and co-infections of 24.7%, 21.6%, and 6.8%, respectively [28]. The findings may suggest that the burden of malaria and helminths co-infections is still a problem in sub-Saharan Africa. HIV infection was not found to be significantly associated with post-partum anemia in our study. This may be explained by early access to anti-retral viral therapy (ART) immediately after diagnosis so as to enable prevention of mother to child transmission [29]. ART given to those women helps in boasting their immunity and down regulate the impact of HIV.

Our study has a number of limitations. First, it was conducted in primary public health care facilities, hence targeted only women who visit health units, missing out those with limited resources for transport, those who may have lost their children at birth and thereafter and those with poor health seeking behaviors. Second, the study missed out women who had died under circumstances where anemia played a role. Third, other factors like cultural and dietary habits were not included in the study and thus a larger, community-based study is recommended as most of Tanzanian women give birth in their home places. Fourth, the nature of study design, being a cross section study, does not show which preceded the other between post-partum anemia and risk factors. Moreover, were unable to determine the last hemoglobin levels of the participants before they gave birth, thus missing the possible major causes of postpartum anemia which include pre-partum anemia. Similarly, convenient sampling instead of random sampling technique was used to obtain study participants. Hence, interpretation of the study findings needs to be done cautiously. Lastly but not least, hemoglobin determination by the kit used in this study may not necessarily reflect the actual current situation.

## Conclusions

The prevalence of post-partum anemia in public primary health care facilities in Dar es Salaam was 34.2%. Absence of a marital partner and less than two years interval between the last two pregnancies were significantly associated with anemia in post-partum period while low parity and delivery by vaginal route were protective. The high prevalence of post-partum anemia found in this study highlights the importance of screening and treating prepartum anemia as well as provision of education on ideal child spacing which may constitute an important approach for reducing the prevalence of anemia.

## Supporting information

S1 File Anonymized dataset underlying the results in this study can be found at Open Science Framework with Identifier: DOI 10.17605/OSF.IO/NXJ9S. (SAV)
